# Ovarian Solid Pseudopapillary Tumor Resembling Benign Hemorrhagic Cyst on Rapid Frozen Section

**DOI:** 10.1155/2020/6473630

**Published:** 2020-05-30

**Authors:** Michelle T. Nguyen, Michael Carter, Zimin Zhao, Alireza Abidi, Melissa Hodeib

**Affiliations:** ^1^Department of Obstetrics and Gynecology, Adventist Health White Memorial, Los Angeles, CA, USA; ^2^Department of Obstetrics and Gynecology, Kaiser Permanente Southern California, Riverside, CA 92505, USA; ^3^Department of Pathology, Kaiser Permanente Southern California, Riverside, CA 92505, USA; ^4^Department of Gynecologic Oncology, Kaiser Permanente Southern California, Riverside, CA 92505, USA

## Abstract

Solid pseudopapillary tumors are rare, with the majority of described cases originating in the pancreas. To date, there are only 10 documented reports of primary ovarian solid pseudopapillary tumors. Here, we describe the case of a 24-year-old woman who presented with worsening pelvic pain and dysmenorrhea. Workup demonstrated a right ovarian solid mass on ultrasound and an elevated serum LDH, which raised concerns for dysgerminoma due to her relatively young age. Therefore, she was taken to the operating room and underwent laparoscopic right salpingo-oophorectomy. On initial rapid frozen section, her ovarian cyst had a grossly hemorrhagic appearance with multiple hemosiderin deposits noted microscopically, which suggested a benign hemorrhagic cyst. However, the final pathology was reported as solid pseudopapillary tumor based on several defining histologic characteristics. Most importantly, immunostaining was positive for *β*-catenin and negative for E-cadherin. This report presents a brief review of the current literature on primary ovarian solid pseudopapillary tumors, including a discussion of expected prognosis after surgical resection, as well as a discussion of the role of immunohistochemistry (IHC) in differentiating ovarian neoplasms in young premenopausal women.

## 1. Introduction

Primary ovarian solid pseudopapillary tumors (SPTs) are rare, with only 10 cases reported in the English literature at the time of this publication [1–8]. SPTs are more commonly found as primary pancreatic tumors. Pancreatic SPTs and ovarian SPTs have overlapping characteristic features on gross appearance and microscopic examination. They both tend to be indolent, and surgical resection generally leads to a very favorable prognosis. One of the key diagnostic features that distinguishes ovarian SPTs from other ovarian tumors is IHC: ovarian SPTs stain positive for nuclear and cytoplasmic *β*-catenin and exhibit a loss of membranous E-cadherin expression.

## 2. Clinical History

**Figure 1:**
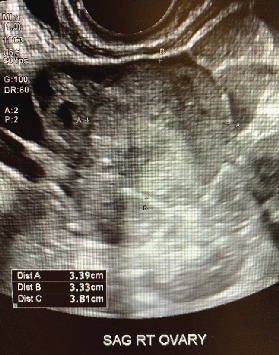
Sagittal view of right ovary on transvaginal ultrasound. The right ovary measures 5.24 cm × 5.52 cm × 3.22 cm. Within the right ovary is a solid heterogenous mass measuring 3.4 cm × 3.3 cm × 3.8 cm.

A 24-year-old African-American nulligravida female presented to a gynecologist with worsening pelvic pain and dysmenorrhea. She was otherwise healthy with regular monthly menses, no medical problems, no changes in weight, and no changes in bladder or bowel habits. Her family history was significant for a paternal grandmother with breast cancer at an unknown age. On physical exam, a palpable mass and tenderness were appreciated in the right adnexa. Pelvic ultrasound showed an enlarged right ovary measuring 5.24 cm × 5.52 cm × 3.22 cm with a solid heterogenous mass measuring 3.4 cm × 3.3 cm × 3.8 cm (Figure 1). This irregular solid tumor demonstrated blood flow (color score < 4) with no papillary structures, ascites, or acoustic shadowing; therefore, it would be classified as “malignant” according to the International Ovarian Tumor Analysis (IOTA) simple ultrasound rules. Computed tomography (CT) scan showed free fluid in the pelvis and a 3.2 cm × 3.4 cm ovoid region of mildly heterogeneous soft tissue density in the right adnexa, consistent with the right ovarian mass seen on ultrasound. Tumor markers were significant for an elevated LDH of 222; however, HCG, AFP, estradiol, sex hormone binding globulin, total and free testosterone, CA-125, AMH, DHA, and inhibin A were all normal. A complex ovarian mass in the setting of elevated LDH and relatively young age raised concerns for dysgerminoma. Therefore, the patient was counseled and ultimately consented for unilateral salpingo-oophorectomy and possible surgical staging if malignancy was suspected on rapid frozen section.

**Figure 2:**
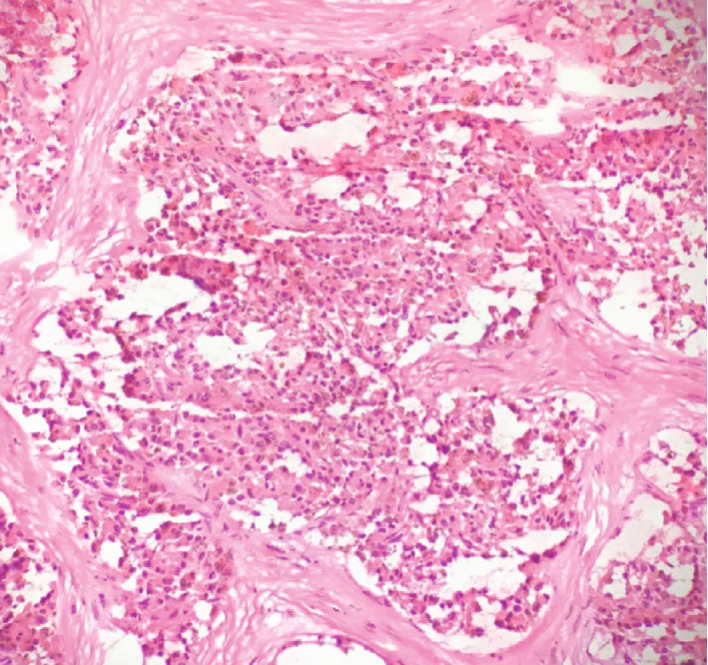
Rapid frozen section of right ovarian mass. There are many tumor cells with appearance mimicking macrophages containing abundant intracellular hemosiderin, suggestive of a hemorrhagic cyst (magnification ×100).

Upon laparoscopic entry into the abdomen, findings included a 5 cm right ovary with a partly solid, partly cystic mass. Otherwise, abdominal and pelvic anatomy appeared normal with no evidence of ascites, carcinomatosis, or metastasis. A right salpingo-oophorectomy was performed without complications. The specimen was removed intact and sent to pathology. On initial rapid frozen section, the ovarian mass grossly appeared very hemorrhagic with multiple hemosiderin deposits microscopically, suggestive of a benign hemorrhagic cyst (Figure 2). Therefore, the surgery was concluded.

**Figure 3:**
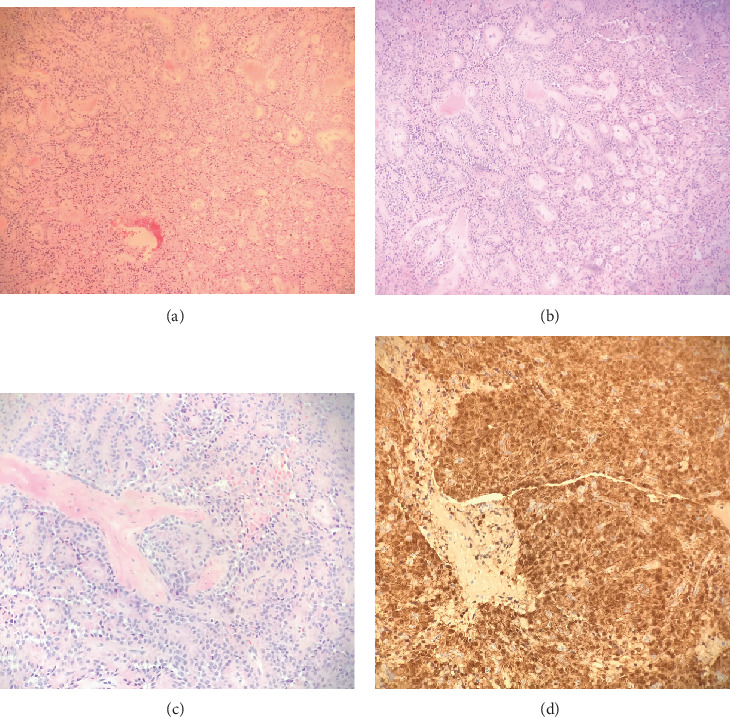
(a) Pseudopapillary growth pattern characteristic of solid pseudopapillary tumors (magnification ×100, H&E). (b) Another view of pseudopapillary growth pattern characteristic of solid pseudopapillary tumors (magnification ×100, H&E). (c) Tumor cells with characteristic eccentric nuclei and extracellular eosinophilic hyaline globules (magnification ×200, H&E). (d) *β*-catenin immunohistochemical staining shows nuclear and cytoplasmic positive staining (magnification ×100, *β*-catenin).

Upon further sampling of permanent sections of the specimen, a diffuse pseudopapillary growth pattern and prominent hyaline globules were noted (Figures 3(a)–3(c)). IHC of tumor cells showed positivity for nuclear and cytoplasmic *β*-catenin and negativity for membranous E-cadherin (Figure 3(d)), consistent with a solid pseudopapillary tumor. IHC of the specimen was also negative for CD31, pancytokeratin, SOX10, inhibin, synaptophysin, and SALL4.

The patient had an uncomplicated postoperative recovery. A postoperative CT scan of the abdomen was ordered to assess for intra-abdominal lesions, particularly in the pancreas. However, the patient became pregnant shortly thereafter and declined to have CT imaging until after delivery. She remains clinically well with resolution of her pelvic pain and continues to have close surveillance with her obstetrician/gynecologist.

## 3. Discussion

Solid pseudopapillary tumors usually arise in the pancreas as a low-grade, indolent neoplasm. In rare cases, pancreatic SPTs may be aggressive and metastasize to the liver or peritoneum, or even more rarely to the ovary [9]. Less than 1% of SPTs are primary extrapancreatic tumors [9], with only 10 cases of primary ovarian SPTs reported to date in the English literature [1–8].

The exact origin of SPTs remains unclear. Some investigators hypothesize that SPTs develop from pluripotent embryonic cells of the pancreas with multipotential differentiation, whereas others suggest that SPTs originate from genital ridge cells which had been attached to pancreatic tissue during early embryogenesis [4, 10]. Primary pancreatic SPTs and primary ovarian SPTs resemble each other both grossly and microscopically. The gross appearance of SPTs is characterized by both cystic and solid components. The defining histologic characteristics of SPTs, as seen in our case, include a pseudopapillary growth pattern, pale eosinophilic cytoplasm, nuclei with fine chromatin, and extracellular hyaline globules (Figures 3(a)–3(c)).

On initial rapid frozen section, the specimen had a grossly hemorrhagic appearance, possibly due to fragmentation within the laparoscopic retrieval bag. Microscopically, a predominance of hemosiderin pigmentation was noted (Figure 2), but not to the extent typically seen in pancreatic SPTs. Therefore, the specimen was intraoperatively diagnosed as a benign hemorrhagic cyst. Our case highlights the importance of IHC in differentiating ovarian masses in young premenopausal women. Differential diagnosis for our patient's ovarian cyst prior to surgery included dysgerminoma due to elevated serum LDH. However, immunostaining was negative for SALL4. Immunostaining was also negative for inhibin (a marker for sex-cord stromal tumors), synaptophysin (a marker for neuroendocrine tumors), SOX10 (a marker for melanoma), and pancytokeratin (a marker for epithelial tumors). The specimen in our case had positive immunostaining for nuclear and cytoplasmic *β*-catenin (Figure 3(d)) and negative immunostaining for membranous E-cadherin, both of which are specifically diagnostic of SPTs.

A comparison of our case to other reported cases of ovarian SPTs revealed several similarities. Ovarian SPTs most frequently occur in young premenopausal women, with an overall age range of 17-57 years. Typical presenting symptoms include abdominal pain, bloating, swelling, and fullness; decreased appetite and weight loss have also been reported. On gross examination, the tumors range in size from 3 cm to 25.5 cm and are usually well-circumscribed masses with both cystic and solid components, although some ovarian SPTs are cystic only. In terms of tumor site, there does not appear to be a predominance of the left ovary versus the right ovary. As with primary pancreatic SPTs, the majority of primary ovarian SPTs have an indolent course, and prognosis is usually very favorable after surgical resection [9]. However, in one exceptional case, metastases of the primary ovarian SPT were noted to the omentum, parametrium, and pelvic lymph nodes; after surgical management (i.e., right salpingo-oophorectomy, total omentectomy, pelvic lymph node dissection, and tumor debulking), the patient remained disease-free on a CT scan 18 months after surgery. In another exception case, the primary ovarian SPT involved the fallopian tube, omentum, cul-de-sac, and abdominal wall; the patient died within 8 months after initial diagnosis despite surgical cytoreduction and adjuvant chemotherapy (3 cycles of carboplatin and paclitaxel followed by 3 cycles of carboplatin and gemcitabine) [4].

Given the rarity of primary ovarian SPTs in the literature, optimal treatment and surveillance remain unclear. In our case, a thorough laparoscopic examination of the abdomen and pelvis revealed no evidence of ascites, carcinomatosis, or metastasis. A CT scan of the abdomen was recommended after surgery to complete evaluation for intraabdominal lesions, particularly in the pancreas. However, the patient conceived shortly after her surgery and declined a CT scan during her pregnancy. Regardless, she is expected to have an indolent course with good prognosis, given that her ovarian SPT histologically did not exhibit as much mitotic activity or necrosis compared to pancreatic SPTs.

## Data Availability

N/A.
